# Editorial: Translational advances in Alzheimer's, Parkinson's, and other dementia: Molecular mechanisms, biomarkers, diagnosis, and therapies, volume II

**DOI:** 10.3389/fnagi.2022.1045828

**Published:** 2022-10-10

**Authors:** Jiehui Jiang, Kuangyu Shi, Yiyun Henry Huang, Chih-Yu Hsu, Kenneth Scott Hettie, Woon-Man Kung

**Affiliations:** ^1^School of Life Science, Institute of Biomedical Engineering, Shanghai University, Shanghai, China; ^2^Department of Nuclear Medicine, Inselspital, Bern University Hospital, University of Bern, Bern, Switzerland; ^3^Department of Informatics, Technical University of Munich, Munich, Germany; ^4^Department of Radiology and Biomedical Imaging, PET Center, Yale University School of Medicine, New Haven, CT, United States; ^5^School of Transportation, Fujian University of Technology, Fuzhou, China; ^6^Molecular Imaging Program at Stanford (MIPS), Department of Radiology, Otolaryngology, Head and Neck Surgery, Stanford University School of Medicine, Palo Alto, CA, United States; ^7^Division of Neurosurgery, Department of Surgery, Taipei Tzu Chi Hospital, Buddhist Tzu Chi Medical Foundation, New Taipei City, Taiwan; ^8^Department of Exercise and Health Promotion, College of Kinesiology and Health, Chinese Culture University, Taipei, Taiwan

**Keywords:** Alzheimer's disease, Parkinson's disease, dementia, neurodegenerative disorders, big data mining, imaging methods, bioinformatic applications

Neurodegenerative dementia, such as Alzheimer's disease (AD), Parkinson's disease (PD), and other dementia has garnered increasing attention (Livingston et al., 2020) from an extensive array of researchers. The application of imaging techniques allows clinicians to explore disease process from micro and/or macro perspectives. Increasing numbers of scientists are also focusing on the relationship between genes and diseases (Jansen et al., 2019). Furthermore, bioinformatics analysis, artificial intelligence methodologies, and molecular imaging techniques have been widely used (Bloem et al., 2021; Buckley, 2021; Li et al., 2021). We can better understand disease mechanisms and etiologies through multidisciplinary approaches, allowing for identification of novel biomarkers for prompt diagnosis. Ultimately, revolutionary treatments for disease management may be developed.

Despite the replacement of some editors in the current Research Topic “*Translational advances in Alzheimer's, Parkinson's, and other dementia: Molecular mechanisms, biomarkers, diagnosis, and therapies, volume II”* by Frontiers in Aging Neuroscience, we aimed to update the advancement of scientific knowledge to improve patient care, considering the anteceding Volume I. After intensive evaluation of numerous international submissions, 57 articles are published in Volume II. We hope this collection will facilitate the gaining of new knowledge and to stimulate a wider application of the available tools for future research. The articles contained in this Research Topic highlight significant insights and innovations in the discipline. These manuscripts can be categorized to the following themes.

## Genetics

Chang et al. explored the relationship across 15 genetic loci, apolipoprotein E4 (*ApoE4*) status, structural covariance networks of gray matter volume, and clinical scale score. Results suggested that AD-susceptible loci interacted with *ApoE4*, structural networks, and are associated with the cognitive outcomes. Xia et al. identified the candidate gene *GJA1*, which was likely to be a target of AD. Fu et al. explored the effect of sex on lipids (cholesterol and low-density lipoprotein) in AD and normal controls with different *APOE* genes. Lüth et al. established a mitochondrial DNA (mtDNA) methylation workflow using Nanopore sequencing and explored its implication in PD. The study of Maraki et al. suggested that there was an association between polygenic risk score and prodromal markers of PD. The accumulation of genetic variants was related to cognitive deficits.

Kuo et al. proposed 87 enriched pathways that are significant for bipolar disorder *via* genetic pathway analytics strategies. In addition, there were 3 subnetworks with multiple hotspots linked with several Gene Ontology processes for bipolar disorder. In another meta-analysis, Bottero et al. found dysregulated genes expression in frontal areas and Brodmann's area 8 for patients with frontotemporal dementia (FTD). They also compared the similarities and differences between sporadic and familial FTD.

## Biomarkers: Molecular

Identification of novel molecular markers for diagnosis and treatment of neurodegenerative disease is important. Using data from Alzheimer's Disease Neuroimaging Initiative (ADNI) cohort, Lin R. R. et al. compared different amyloid-beta (Aβ), tau, and neurodegeneration (AT(N)) biomarkers with cognitive progression. The biomarkers included cerebrospinal fluid (CSF) Aβ42, amyloid positron emission tomography (PET) ([18F]flutemetamol), phosphorylated tau (p-tau), tau PET ([18F]flortaucipir), total tau (t-tau), hippocampal volume, temporal cortical thickness, [18F]fluorodeoxyglucose (FDG) PET, and plasma neurofilament light (NfL). Margraf et al. evaluated the biomarkers in CSF of patients with probable cerebral amyloid angiopathy, including Aβ40, Aβ42, t-tau, and phosphorylated tau 181 (p-tau^181^). Xu X. et al. analyzed 31 AD patients with ^18^F-APN-1607 PET imaging. This tracer has high binding affinity for 3- and 4-repeat tau deposits. Data showed that this tracer is effective to evaluate change pattern of tau protein deposition in AD patients. Ezura et al. categorized patients with corticobasal syndrome (CBS), progressive supranuclear palsy (PSP), and AD based on ^18^F-THK5351 PET. They reported sites including precentral gyrus and inferior temporal gyrus, which were susceptible to disease-related pathologies.

Using immunofluorescence-based assays, Puentes et al. explored if Poly (ADP-ribose) Polymerase-1 (PARP-1) enzymatic product (PAR) promotes the aberrant cytoplasmic accumulation of hyperphosphorylated (S129) forms of alpha-synuclein (pαSyn) in transgenic murine and post mortem PD/Parkinson's disease dementia (PDD) patient samples. Liao P. H. et al. reported an increased colonic leucine-rich repeat kinase 2 (LRRK2) expression in PD patients, where the level was related to disease severity. Nguyen et al. proposed that the mitochondrial protein Miro1 is a promising molecular marker. It has potential to detect both PD and at-risk populations. Scholefield et al. measured the amount of brain urea in PD patients. Increased urea level provides an understanding about the pathogenic mechanism in PD patients with dementia.

## Biomarkers: Signaling and imaging

On the basis of quantitative electroencephalography, Novak et al. used a wavelet-transform based time-frequency algorithm to assess the instantaneous predominant frequency (IPF) at 60 ms intervals. Results demonstrated that there was an altered relative time spent by the IPF in cognitively impaired PD individuals.

For diagnosis of AD and its prodromal stages, Ho T. K. K. et al. validated the capability of functional near-infrared spectroscopy. They further used deep learning models to do the multi-class classification. The Convolutional Neural Network-Long Short-Term Memory (CNN-LSTM) model obtained the highest accuracy.

Shen et al. enrolled 27 multiple system atrophy (MSA) with predominant parkinsonism (MSA-P) patients and 57 MSA with predominant cerebellar ataxia (MSA-C) patients. They found a relationship between ^18^F-FDG uptake and neuropsychological scores, especially in frontal lobe and cerebellum. Ba et al. assessed stereopsis and eye movement abnormalities in PD using 2D and Titmus stereotests, followed by 3D active shutter system. They suggested that visual parameters may potentially serve as the clinical biomarkers for PD. Amboni et al. reported that the gait pattern recorded by digital cameras was associated with mild cognitive impairment (MCI) patients of PD. Shang et al. compared the neurovascular decoupling index between PD with normal cognition (NC) and PD with MCI, including global and regional cerebral blood flow-regional homogeneity (CBF-ReHo) correlation coefficients and CBF-ReHo ratios. Results showed regionally specific neurovascular decoupling in PD-MCI, was most prominent in visual-spatial areas. Hu Q. et al. focused on the metrics of intrinsic brain activity in MCI patients who revert to AD, including amplitude of low-frequency of fluctuation (ALFF), fraction amplitude of low-frequency fluctuation (fALFF), ReHo, and degree centrality (DC). Changed activity of these MCI reverters helps us to explore AD. Khatri et al. provided a diagnostic framework to identify AD and to differentiate whether MCI will be converted to AD. They used biomarkers obtained from resting-state functional magnetic resonance imaging (rs-fMRI), including ALFF, fALFF, ReHo, DC, and salience networks (SN).

To investigate the relationship between Aβ and cognitive decline in AD, Li B. et al. performed a simultaneous memory task functional MRI and amyloid PET experiment. They reported a decreased functional connectivity during the memory retrieval stage. There was a negative correlation between Aβ deposition and connectivity. Moreover, functional connectivity mediated an adverse effect of Aβ on memory. Yun et al. reported the relationship across plasma Aβ analysis, amyloid PET scanning, and neuropsychological tests. Zheng et al. focused on patients with vascular dementia caused by aneurysmal subarachnoid hemorrhage (aSAH). They investigated the topological characteristics of brain network. Results discovered a decreased characteristic path length in the aSAH patients. Xue et al. analyzed dynamic functional connectivity (DFC) based on the sliding time-window correlation. They found significant alteration of DFC in brain between subjective cognitive decline (SCD) and amnestic mild cognitive impairment (aMCI), which could be applied for precise preclinical AD diagnosis. Xing et al. used time-sliding window method to explore the spatiotemporal evolution of brain functional networks. It could provide information about abnormalities of the functional organization of AD patients. Tian et al. also used the sliding time window approach to study brain activity of PD patients.

## Computer-aided diagnosis/image processing

Xu J. et al. developed a computer-aided classification framework, which could be used to identify PD, multiple system atrophy (MSA), and PSP. This framework consisted of MRI-assisted PET segmentation, feature extraction and prediction, and automatic subject classification. Smith et al. used the Statistical Parametric Mapping technique to create a database, which enhanced the diagnostic performance in AD through its quantitative approach.

Seo et al. provided an approach based on deep learning, which could generate brain parenchyma mask and target volumes-of-interest (VOIs) for mouse brain to solve the problem about skull-stripping and brain region segmentation of PET images. Cai et al. proposed an Elastomeric UNet (EUNet) structure for medical image segmentation. It performed well on a self-built dataset and publicly benchmark retinal datasets.

## Therapies

Pan et al. found that acupuncture could regulate the peripheral immune function and inflammation of the vascular dementia rats. Additionally, it could improve the cognitive dysfunction of the rats. Shyu et al. explored the effect of methamphetamine to enhance cognitive function or to alleviate AD symptoms in rats. Lin L. et al. investigated the mechanisms of tourette syndrome (TS) in a mice model. Outcomes revealed that substantia nigra pars compacta (SNpc) and dorsal striatum (dSTR) may be the targets for treatment. A research from Ko, Xu, Lo., et al. reported that alpha-lipoic acid (ALA) could ameliorate cognition impairment in high-fat diet (HFD) plus streptozotocin (STZ) induced diabetic rats.

Another paper by Ko, Xu, Chang., et al. investigated the enhancing effect of vitamin-like ALA on phagocytosis of oligomeric amyloid-beta (oAβ_1 − 42_) in mouse microglial cell lines. Chiang et al. reported that 7, 8-dihydroxyflavone (7, 8-DHF), quercetin, and apigenin targeting on heat shock protein family B (small) member 1 (HSPB1), nuclear factor erythroid 2-like 2 (NRF2), and tropomyosin-related kinase B (TRKB) could reduce tau aggregation and protect cell lines against tau neurotoxicity, which may be used to treat AD.

Liu et al. reported that *Lycium barbarum* extracts (LBE) may be a potential neuroprotectant for AD through synapse preservation through a brief research report. Shawki et al. revealed the role of an antidiabetic drug liraglutide in Huntington's disease (HD).

Lin W. et al. investigated PD patients who underwent subthalamic nucleus deep brain stimulation (STN-DBS). They found that the levodopa (L-DOPA) challenge test could not predict the quality of life (QoL) outcomes after implantation surgery. However, it could be incorporated into a regression prediction model to predict the effect of DBS. Barcia et al. reported one case about DBS surgery on AD. They assessed the safety of DBS in this AD patient using the fornix as stimulation target. Except for verbal fluency, the cognition of patient seems to return to baseline level after 24 months stimulation. Combined with machine learning, Phokaewvarangkul et al. used a Parkinson's glove and applied electrical muscle stimulation (EMS) for resting tremor during “on” period. They concluded that the LSTM model had the highest accuracy for predicting pulse amplitude which elicited the longest tremor reset time.

## Reviews

There are 8 crucial reviews included in this Research Topic. As neuroinflammation is a hallmark for the progression of AD and PD, Ponce et al. reviewed the anti-inflammatory and pro-resolving effects of polyunsaturated fatty acid (PUFA)-derived mediators (Specialized Pro-resolving Mediators–SPM) in neurodegenerative disorders. Hu S. et al. reviewed studies discussing the association of soluble triggering receptor expressed on myeloid cells 2 (sTREM2) levels in CSF and AD risk. They also provided upcoming recommendations. Bandopadhyay et al. discussed the molecular mechanisms and therapeutic approaches for L-DOPA-induced dyskinesia in PD patients. Although metformin is a first-line medication for Type 2 Diabetes Mellitus (T2DM), it also showed beneficial effects on neurodegenerative disorders. Liao W. et al. reviewed the mechanisms of metformin in AD pathology. Mari and Mestre. provided a mini review about the PD modification clinical trials and discussed what to do in the future.

Cao et al. reviewed the recently developed PET tracers for evaluation of pathophysiology in tauopathy animal models. They also discussed several considerations for future tauopathy animal studies. Hou and Shang reviewed the current biomarkers based on MRI for cognitive impairment in PD.

Fujikawa et al. reviewed the development of invasive and non-invasive medical devices to alleviate motor symptoms in PD.

## Miscellaneous

Yang et al. explored the clinical application of the sum of boxes of the Clinical Dementia Rating (CDR) Scale. They found that it could be used to determine SCD, MCI, and dementia. Zhou et al. assessed the prevalence and clinical features of autonomic dysfunction in Chinese patients with PD. This study included a large multicenter cohort of 2,556 individuals. For postoperative delirium (POD), Li K. et al. reported the anti-inflammatory and blood-brain barrier (BBB) protective effects of Netrin-1 (NTN-1) (an axonal guidance molecule) in an inflammatory environment in mice.

To explore the association between gallstone disease (GD) and atrial fibrillation (AF), Ho T. C. et al. focused on a longitudinal large scale population-based cohort and reported that GD patients had an increased AF risk. Furthermore, cholecystectomy was related to a reduction of AF risk. Zhao et al. developed an intelligent healthcare system that could hide patients' confidential data into electrocardiogram (ECG) signals.

## Author contributions

JJ and KS wrote the draft. KSH copyedited for the language. YHH, C-YH, and W-MK reviewed and revised the manuscript. All authors listed have made a substantial, direct, and intellectual contribution to the work and approved it for publication.

## Conflict of interest

The authors declare that the research was conducted in the absence of any commercial or financial relationships that could be construed as a potential conflict of interest.

## Publisher's note

All claims expressed in this article are solely those of the authors and do not necessarily represent those of their affiliated organizations, or those of the publisher, the editors and the reviewers. Any product that may be evaluated in this article, or claim that may be made by its manufacturer, is not guaranteed or endorsed by the publisher.
